# Prevalence and co‐occurrence of symptoms of mental and substance use disorders among people with HIV age 40 and older in low‐ and middle‐income countries: a cross‐sectional study

**DOI:** 10.1002/jia2.26359

**Published:** 2024-10-07

**Authors:** Angela M. Parcesepe, Melissa Stockton, Charlotte Bernard, Tukiya Kanguya, Edith Kamaru Kwobah, Alvaro Lopez, Gad Murenzi, Jeremy Ross, Albert Minga, Fernanda Maruri, Mpho Tlali, Suzanne Goodrich, Hugo Perazzo, Françoise Musabyimana, Smita Nimkar, Kathryn Lancaster, IeDEA Consortium

**Affiliations:** ^1^ Department of Maternal and Child Health Gillings School of Global Public Health University of North Carolina at Chapel Hill Chapel Hill North Carolina USA; ^2^ Carolina Population Center University of North Carolina at Chapel Hill Chapel Hill North Carolina USA; ^3^ Department of Epidemiology Gillings School of Global Public Health University of North Carolina at Chapel Hill Chapel Hill North Carolina USA; ^4^ University of Bordeaux, National Institute for Health and Medical Research (INSERM), Research Institute for Sustainable Development (IRD), Bordeaux Population Health Research Centre, UMR 1219, Team GHiGS Bordeaux France; ^5^ Center for Infectious Disease and Research in Zambia Lusaka Zambia; ^6^ Department of Mental Health Moi Teaching and Referral Hospital Eldoret Kenya; ^7^ Departamento de Infectología Instituto Nacional de Ciencias Médicas y Nutrición “Salvador Zubirán” Mexico City Mexico; ^8^ Rwanda Military Hospital and Research for Development (RD Rwanda) Kigali Rwanda; ^9^ TREAT Asia/amfAR – The Foundation for AIDS Research Bangkok Thailand; ^10^ Centre Médical de Suivi des Donneurs de Sang/CNTS Abidjan Côte d'Ivoire; ^11^ Vanderbilt University Nashville Tennessee USA; ^12^ Centre for Infectious Disease Epidemiology & Research (CIDER) School of Public Health & Family Medicine University of Cape Town Cape Town South Africa; ^13^ Division of Infectious Diseases Indiana University School of Medicine Indianapolis Indiana USA; ^14^ National Institute of Infectious Diseases Evandro Chagas‐Oswaldo Cruz Foundation (INI/FIOCRUZ) Rio de Janeiro Brazil; ^15^ B J Government Medical College Clinical Research Site Pune India; ^16^ Wake Forest University Winston‐Salem North Carolina USA

**Keywords:** mental health, substance use, HIV, LMICs, depression, anxiety

## Abstract

**Introduction:**

Due to the increased effectiveness of and access to antiretroviral therapy (ART), people with HIV (PWH) are living longer. As a result, the population of older PWH has increased. Mental and substance use disorders (MSDs) are common and frequently co‐occurring among PWH and are associated with poor HIV care outcomes. Research into the prevalence and co‐occurrence of MSDs among ageing PWH remains limited, particularly in low‐ and middle‐income countries (LMICs).

**Methods:**

We analysed data collected between 2020 and 2022 from the International epidemiology Databases to Evaluate AIDS (IeDEA) Sentinel Research Network cohort of PWH aged 40 years or older on ART at 11 HIV clinics in Brazil, Côte d'Ivoire, India, Kenya, Mexico, Uganda, Rwanda, Togo, Vietnam, Zambia and Zimbabwe. We estimated the prevalence and co‐occurrence of unhealthy alcohol use (AUDIT‐C ≥3 for women, ≥4 for men), unhealthy drug use (ASSIST >3 for cannabis, cocaine, amphetamines, inhalants, sedatives, hallucinogens and/or opioids), and moderate to severe symptoms of depression (PHQ‐9 ≥10), anxiety (GAD‐7 ≥10) and post‐traumatic stress disorder (PTSD) (PCL‐5 ≥33). Psychiatric multimorbidity was defined as having symptoms of two or more disorders assessed. Log binomial models assessed the association between socio‐demographic and HIV care characteristics and symptoms of anxiety, depression, PTSD or unhealthy substance use.

**Results:**

Of 2821 participants, the prevalence of unhealthy alcohol and drug use was 21% and 5%, respectively. The prevalence of moderate to severe symptoms of depression, anxiety and PTSD was 14%, 9% and 6%, respectively. Overall, the prevalence of psychiatric multimorbidity was 11%. Among those with symptoms of at least one mental health or substance use outcome assessed (*n* = 1036), the prevalence of psychiatric multimorbidity was 31%. In binomial models, the prevalence of symptoms of depression and anxiety was higher, while the prevalence of unhealthy alcohol and drug use was lower among women than men.

**Conclusions:**

Unhealthy alcohol use and symptoms of depression were most commonly reported, among this cohort of PWH aged 40 or older across 11 LMICs. Integration of MSD screening and treatment into HIV care should be prioritized. The effectiveness and implementation of transdiagnostic or multi‐focus mental health treatment approaches in HIV care settings should be examined.

## 1 INTRODUCTION

Due to the increased effectiveness of and access to antiretroviral therapy (ART), people with HIV (PWH) are living longer [1]. With early ART initiation, continued ART adherence and sustained viral suppression, current ART treatment regimens can afford PWH a similar life expectancy to individuals without HIV. As a result, the life expectancy of PWH has increased over the past decades in high‐ and low‐ and middle‐income countries (LMICs) [2, 3, 4]. As HIV has become a chronic health condition, the population of older PWH (typically defined as individuals aged ≥50 years) has increased [5]. UNAIDS has estimated that the number of PWH aged 50 and older increased from approximately 5 million in 2015 to approximately 8 million in 2020 [6].

With a longer life expectancy, the risk for non‐communicable diseases (NCDs) among PWH has increased. Mental and substance use disorders (MSDs) are among the most common NCDs among PWH. It has been estimated that 28−62% of PWH have symptoms of one or more mental or substance use disorders [8]. MSDs have been associated with suboptimal HIV care continuum outcomes, including late HIV diagnosis, suboptimal ART adherence and virologic failure [9, 10, 11, 12]. Among the general population, psychiatric multimorbidity, the presence of two or more co‐occurring mental or substance use disorders, is common and has been associated with greater psychiatric symptom severity and worse mental health outcomes [14, 15, 16, 17, 18, 19]. Data on the prevalence, co‐occurrence and correlates of MSDs among older PWH in resource‐limited settings remain scarce. As the population of older PWH increases, greater understanding of the prevalence and co‐occurrence of MSD among ageing PWH can inform strategies to address mental health and substance use screening and treatment needs as PWH age across resource‐constrained settings.

The International epidemiology Databases to Evaluate AIDS (IeDEA) Consortium is an international research consortium funded by the U.S. National Institutes of Health that collects observational HIV care and treatment data across global settings [20, 21, 22]. IeDEA established the Sentinel Research Network (SRN), a longitudinal cohort of PWH to advance understanding of the prevalence and impact of NCDs including cardiovascular disease, MSDs and liver disease among ageing PWH in LMICs. Given the limited information about the incidence, prevalence and impact of these NCDs among PWH in LMICs, the IeDEA Consortium set the eligibility criteria for participation in the IeDEA SRN cohort as aged 40 or older. While age 50 and older is the typical definition of “older” PWH, most existing research on older PWH has been conducted with PHW in high‐income countries. Thus, it remains unclear what the optimal age‐related cut‐off should be when studying NCDs among PWH in LMICs.

The objectives of the current analysis are to (1) estimate the prevalence and co‐occurrence of symptoms of depression, anxiety and post‐traumatic stress disorder (PTSD), and unhealthy substance use and (2) assess the relationship between socio‐demographic and HIV care characteristics and symptoms and co‐occurrence of symptoms of anxiety, depression, PTSD or unhealthy substance use among PWH 40 years or older enrolled in the IeDEA SRN.

## 2 METHODS

### 2.1 Study design

This analysis uses baseline data from the IeDEA‐SRN cohort which collects longitudinal data on risk factors and prevalence of NCDs, including symptoms of MSDs. The SRN is a cohort nested in six geographic regions of the IeDEA Consortium: the Caribbean, Central, and South America (CCASAnet), the Asia‐Pacific, and West, East, Central, and Southern Africa [21, 23]. IeDEA‐SRN participants were recruited during routine clinical care visits. Participants in this analysis were enrolled in the IeDEA‐SRN between October 2020 and 2022.

### 2.2 Study sites and population

This analysis includes data from 11 IeDEA SRN research sites located in Brazil, Côte d'Ivoire, India, Kenya, Mexico, Rwanda, Togo, Uganda, Vietnam, Zambia and Zimbabwe. All SRN sites recruited PWH ≥40 years who had initiated ART ≥6 months prior to study enrolment. SRN study sites sought to recruit a representative sample of eligible PWH at their sites.

### 2.3 Measures

#### 2.3.1 Unhealthy alcohol or drug use and moderate to severe symptoms of anxiety, depression and PTSD

Trained study staff screened IeDEA‐SRN participants for unhealthy alcohol or drug use and symptoms of anxiety, depression and PTSD. Depressive symptoms were assessed with the Patient Health Questionaire‐9 (PHQ‐9). Scores of ≥10 were categorized as moderate or severe depressive symptoms [24, 25, 26, 27, 28, 29, 30]. Anxiety symptoms were assessed with the General Anxiety Disorder‐7 (GAD‐7). Scores of ≥10 were categorized as moderate or severe anxiety symptoms [27, 31, 32]. PTSD was assessed with the PTSD Checklist for the DSM‐5 (PCL‐5). Scores of ≥33 were categorized as symptoms of probable PTSD [33]. Alcohol use was assessed with the Alcohol Use Disorders Identification Test‐C (AUDIT‐C). Scores of ≥3 for women and ≥4 for men were categorized as unhealthy alcohol use [34, 35]. Drug use was assessed with the Alcohol, Smoking and Substance Involvement Screening Test (ASSIST). ASSIST‐specific substance involvement scores >3 for cannabis, cocaine, amphetamines, inhalants, sedatives, hallucinogens or opiates were categorized as unhealthy drug use [36, 37].

The PHQ‐9 has been previously validated in Brazil, Rwanda, India, Kenya, Mexico, Uganda and Zimbabwe [38, 39, 40, 41, 42, 43, 44]. The GAD‐7 has been previously validated in Brazil, Kenya and Zimbabwe [43, 45, 46]. The PCL‐5 has been previously validated in Brazil, Rwanda, Mexico and Zimbabwe [47, 48, 49, 50]. The AUDIT has been previously validated in Brazil, Mexico, Zambia, and Uganda [51, 52, 53, 54]. The ASSIST has been previously validated in Brazil, India, Mexico, Zambia and Zimbabwe [55, 56, 57, 58]. Screening tools that had been previously translated and validated in the relevant local languages were used without modification. If a screening tool had not been previously validated in the relevant local language, the tool was modified using the following process. First, a local translator with HIV, medical or public health experience completed an initial translation of the instrument. Following this initial translation, HIV clinic staff with relevant clinical or research experience reviewed the translated instruments. The local translator and site staff reviewed the translation and made any necessary adjustments by consensus. In addition, cognitive interviews were conducted at each SRN site. Reports from cognitive interviews were shared with the SRN investigators and study staff to ensure contextual modifications did not interfere with the replicability of instruments.

#### 2.3.2 HIV care history

HIV care variables were ascertained through routinely collected IeDEA data. Years on ART was calculated as the total number of years between the date of IeDEA‐SRN enrolment and the date of first ART initiation. Having ever been diagnosed with AIDS was also captured in routinely collected IeDEA data which asked whether the participant had ever been given an AIDS diagnosis.

The following socio‐demographic information was collected at SRN enrolment: age, sex at birth, marital status, education and monthly household income.

### 2.4 Analysis

Univariate analyses were conducted to assess the prevalence of symptoms of moderate to severe depression, moderate to severe anxiety, probable PTSD, unhealthy alcohol use and unhealthy drug use, overall as well as by country and sex. Separate bivariable log binomial models were used to investigate the relationship between socio‐demographic or HIV care factors and the prevalence and co‐occurrence of mental health or substance use outcomes.

Missing mental health or substance use data were minimal. However, for each instance of missing mental health or substance use data, we assessed whether missing responses could potentially impact the categorization of the mental health or substance use outcome. Participants with missing response(s) that could not impact the categorization of the mental health or substance use outcome were included. Those with missing response(s) that could have impacted the categorization of the mental health or substance use outcome were excluded from the analysis. All IeDEA‐SRN data were captured and managed in REDCap [59, 60]. All analyses were completed using STATA 17.

### 2.5 Ethical considerations

The study protocol and data collection instruments were reviewed and approved by each SRN site, regional coordinating centre and regional data management centre IRB prior to study implementation. All participants provided written informed consent.

## 3 RESULTS

The 2821 participants ranged in age from 40 to 85, with nearly half (49%) between 40 and 49 years of age (Table 1). Over half (55%) of participants were women, 52% were married or living with a partner and 40% had not attained a secondary level of education. Overall, 42% lived on a monthly income of less than $80 USD. Participants had been on ART for an average of 11 years and 44% of participants had been previously diagnosed with AIDS.

**Table 1:** Participant characteristics (*N* = 2821)

Characteristic	Mean (Standard deviation) or *n* (%)
Age (Range: 40−85)	51 (8)
40−49	1375 (49)
50−59	1044 (37)
≥60	402 (14)
Sex	
Male	1256 (45)
Female	1565 (55)
Marital status	
Single	476 (17)
Married or living with partner	1472 (52)
Widowed, separated or divorced	869 (31)
Education	
Primary or less	1119 (40)
Secondary	1271 (45)
Tertiary	409 (15)
Monthly income (USD)	
< 80	1152 (42)
80−200	702 (26)
> 200	877 (32)
Country	
India	200 (7)
Brazil	225 (8)
Rwanda	599 (21)
Kenya	200 (7)
Zambia	198 (7)
Cote D'Ivoire	300 (11)
Mexico	199 (7)
Uganda	100 (3)
Zimbabwe	300 (11)
Vietnam	200 (7)
Togo	300 (11)
HIV care characteristics	
Years on ART (Range: 0.5−30.3)	11.3 (5)
Ever diagnosed with AIDS	1031 (44)

Missingness by variable: Marital status *n* = 4; Education *n* = 22; Income *n* = 90; Years on ART *n* = 22; Ever diagnosed with AIDS *n* = 457.

### 3.1 Prevalence of symptoms of anxiety, depression, PTSD and unhealthy alcohol or drug use

The prevalence of moderate to severe symptoms of depression and anxiety was 14% and 9%, respectively (Table 2). Overall, 6% of participants screened positive for symptoms of probable PTSD. The prevalence of unhealthy alcohol use and unhealthy drug use was 21% and 5%, respectively. Overall, 37% of participants screened positive for symptoms of depression, anxiety, PTSD, or unhealthy alcohol or drug use. Among the entire sample (*n* = 2821), the prevalence of psychiatric multimorbidity—symptoms of more than one mental health or substance use outcome assessed was 11%. Among those with symptoms of at least one mental health or substance use outcome assessed (*n* = 1036), the prevalence of psychiatric multimorbidity was 31%. The prevalence of psychiatric multimorbidity was 28% and 58% among those with symptoms of unhealthy alcohol or drug use, respectively. The prevalence of psychiatric multimorbidity was 91% among those with symptoms of probable PTSD.

**Table 2:** Prevalence and co‐occurrence of symptoms of depression, anxiety, PTSD and unhealthy alcohol or drug use

*n* (%)	Total (*N* = 2821)	Depression (*n* = 387)	Anxiety (*n* = 262)	PTSD (*n* = 159)	Unhealthy alcohol use (*n* = 599)	Unhealthy drug use (*n* = 132)
Symptoms of common mental disorders						
Depression	387 (14)		172 (66)	109 (69)	91 (15)	23 (17)
Anxiety	262 (9)	172 (44)		102 (64)	71 (12)	22 (17)
PTSD	159 (6)	109 (28)	102 (39)		57 (9)	20 (15)
**Symptoms depression, anxiety or PTSD**	512 (18)				131 (22)	39 (30)
**Number of common mental disorders**						
1	303 (11)	193 (50)	75 (29)	35 (22)	67 (11)	19 (14)
2	122 (4)	107 (28)	100 (38)	37 (23)	40 (7)	14 (11)
3	87 (3)	87 (22)	87 (33)	87 (55)	24 (4)	6 (4)
Substance use						
Unhealthy alcohol use	599 (21)	91 (24)	71 (27)	57 (36)		56 (42)
Unhealthy drug use	132 (5)	23 (6)	22 (8)	20 (13)	56 (9)	
Unhealthy alcohol or drug use	675 (24)	104 (27)	83 (32)	64 (40)		
**Psychiatric multimorbidity**	323 (11)	233 (60)	205 (78)	144 (91)	168 (28)	76 (58)

Missingness by variable Depression *n* = 3; Anxiety *n* = 4; PTSD *n* = 3; Unhealthy alcohol use *n* = 4; Unhealthy drug use *n* = 1; Symptoms of depression, anxiety or PTSD *n* = 1.

Abbreviation: PTSD, post‐traumatic stress disorder.

### 3.2 Associations between socio‐demographic and HIV care characteristics and symptoms of depression, anxiety, PTSD and unhealthy substance use

The prevalence of moderate to severe symptoms of anxiety, probable PTSD and unhealthy alcohol use was lower among those aged 60 or older as compared to those aged 40−49 (Tables 3 and 4) (anxiety: 6% vs. 10%, prevalence ratio [PR] 0.64 [95% CI 0.43−0.96]; PTSD 3% vs. 6%, PR 0.50 [95% CI 0.28−0.89]; unhealthy alcohol use: 13% vs. 23%, PR 0.58 [95% CI 0.44−0.75]). The prevalence of moderate to severe symptoms of depression and anxiety was higher among women than men (depression: 17% vs. 10%, PR 1.68 [95% CI: 1.37−2.05]; anxiety: 10% vs. 8%; PR 1.28 [95% CI: 1.01−1.62]), while the prevalence of unhealthy alcohol use (15% vs. 29%; PR 0.52 [95% CI: 0.45−0.60]) and unhealthy drug use (3% vs. 7%; PR 0.35 [95% CI: 0.24−0.50]) was lower among women compared to men. The prevalence of moderate to severe symptoms of depression (9% vs. 18%; PR 0.51 [95% CI: 0.42−0.61]), moderate to severe symptoms of anxiety (7% vs. 12%; PR 0.56 [95% CI: 0.44−0.72]), symptoms of probable PTSD (3% vs. 8%; PR 0.41 [95% CI: 0.29−0.57]), unhealthy drug use (4% vs. 6% PR 0.67 [95% CI 0.48, 0.94]) and psychiatric multimorbidity (8% vs. 15%, PR 0.58 [95% CI 0.47−0.71]) were all lower among those who were married or partnered compared to those who were not. The prevalence of symptoms of all MSDs assessed varied substantially by country. For example, the prevalence of moderate to severe symptoms of depression ranged from 2% among participants in Vietnam to 32% among participants in Brazil, while the prevalence of unhealthy drug use ranged from 1% among participants in India to 23% among participants in Mexico. The prevalence of symptoms of probable PTSD (5% vs. 7%; PR 0.70 [95% CI 0.52−0.95]) and unhealthy alcohol use (18% vs. 27%; PR 0.66 [95% CI 0.58, 0.77]) was lower among those who had been on ART for 10 years or more compared to those who had been on ART for less than 10 years. The prevalence of moderate to severe symptoms of anxiety was higher among those who had been previously diagnosed with AIDS compared to those who had never been diagnosed with AIDS (12% vs. 8%; PR 0.67 95% CI 0.52, 0.86). The prevalence and co‐occurrence of unhealthy substance use and moderate to severe symptoms of depression, anxiety and PTSD, stratified by gender, age, marital status and educational attainment are presented in the Supplementary Appendix.

**Table 3:** Association between socio‐demographic characteristics or HIV care history and symptoms of mental health or substance use disorders

*n* (row%)	Total (*N* = 2821) *N* (col %)	Depression (*N* = 387) *N* (row %)	Anxiety (*N* = 262) *N* (row %)	PTSD (*N* = 159) *N* (row %)	Unhealthy alcohol use (*N* = 599) *N* (row %)	Unhealthy drug use (*N* = 132) *N* (row %)	Psychiatric multimorbidity (*N* = 323) *N* (row %)
**Age (Range: 40**−**85)**							
40−49	1375 (49)	193 (14)	139 (10)	88 (6)	321 (23)	72 (5)	177 (13)
50−59	1044 (37)	151 (14)	97 (9)	58 (6)	224 (21)	43 (4)	119 (11)
≥60	402 (14)	43 (11)	26 (6)	13 (3)	54 (13)	17 (4)	27 (7)
**Sex**							
Men	1256 (45)	125 (10)	101 (8)	72 (6)	364 (29)	92 (7)	149 (12)
Women	1565 (55)	262 (17)	161 (10)	87 (6)	235 (15)	40 (3)	174 (11)
**Marital status**							
Not married or living with partner	1345 (48)	249 (18)	162 (12)	110 (8)	278 (21)	76 (6)	198 (15)
Married or living with partner	1472 (52)	138 (9)	100 (7)	49 (3)	320 (22)	56 (4)	125 (8)
**Education**							
Primary or less	1119 (40)	175 (16)	119 (11)	59 (5)	203 (18)	43 (4)	127 (11)
Secondary or greater	1680 (60)	209 (12)	140 (8)	99 (6)	391 (23)	89 (5)	193 (11)
**Monthly income (USD)**							
< 80	1152 (42)	190 (16)	111 (10)	66 (6)	199 (17)	37 (3)	132 (11)
80−200	702 (26)	81 (12)	52 (7)	36 (5)	145 (21)	25 (4)	69 (10)
> 200	877 (32)	101 (12)	87 (10)	51 (6)	234 (27)	63 (7)	109 (12)
**Country**							
India	200 (7)	9 (4)	7 (3)	1 (1)	5 (2)	1 (1)	4 (2)
Brazil	225 (8)	72 (32)	63 (28)	28 (12)	71 (32)	16 (7)	65 (29)
Rwanda	599 (21)	84 (14)	49 (8)	31 (5)	95 (16)	2 (0)	51 (8)
Kenya	200 (7)	8 (4)	2 (1)	3 (2)	17 (8)	0 (0)	3 (2)
Zambia	198 (7)	38 (19)	16 (8)	18 (9)	82 (41)	6 (3)	37 (19)
Cote D'Ivoire	300 (11)	48 (16)	50 (17)	22 (7)	78 (26)	3 (1)	38 (13)
Mexico	199 (7)	35 (18)	29 (15)	26 (13)	48 (24)	46 (23)	45 (23)
Uganda	100 (3)	13 (13)	17 (17)	0 (0)	24 (24)	1 (1)	12 (12)
Zimbabwe	300 (11)	37 (12)	13 (4)	10 (3)	42 (14)	8 (3)	21 (7)
Vietnam	200 (7)	3 (2)	0 (0)	0 (0)	46 (23)	2 (1)	1 (1)
Togo	300 (11)	40 (13)	16 (5)	20 (7)	91 (30)	47 (16)	46 (15)
**Years on ART**							
< 10 Years	1072 (38)	162 (15)	107 (10)	74 (7)	285 (27)	60 (6)	154 (14)
≥ 10 Years	1727 (62)	222 (13)	154 (9)	84 (5)	305 (18)	69 (4)	166 (10)
**Diagnosed with AIDS**							
Never	1333 (56)	179 (13)	104 (8)	71 (5)	250 (19)	49 (4)	139 (10)
Ever	1031 (44)	150 (15)	121 (12)	58 (6)	215 (21)	38 (4)	122 (12)

Missingness by variable: Marital status *n* = 4; Education *n* = 22; Income *n* = 90; Years on ART *n* = 22; Ever diagnosed with AIDS *n* = 457; Depression *n* = 3; Anxiety *n* = 4; PTSD *n* = 3; Unhealthy alcohol use *n* = 4; Unhealthy drug use *n* = 1.

**Table 4:** Prevalence ratios of socio‐demographic or HIV care characteristics and symptoms of mental health or substance use disorders

Characteristic (referent)	Prevalence ratios (95% CIs)					
	Depression	Anxiety	PTSD	Alcohol use	Drug use	Psychiatric multimorbidity
Age						
50−59 years (40−49)	1.03 (0.84−1.26)	0.91 (0.72−1.18)	0.87 (0.63−1.20)	0.92 (0.79−1.07)	0.79 (0.54−1.14)	0.78 (0.64−0.96)
60+ years (40−49)	0.76 (0.56−1.04)	0.64 (0.43−0.96)	0.50 (0.28−0.89)	0.58 (0.44−0.75)	0.81 (0.48−1.36)	0.55 (0.38−0.80)
Female (Male)	1.68 (1.37−2.05)	1.28 (1.01−1.62)	0.97 (0.72−1.31)	0.52 (0.45−0.60)	0.35 (0.24−0.50)	0.94 (0.76−1.15)
Married/partnered (Not married/partnered)	0.51 (0.42−0.61)	0.56 (0.44−0.72)	0.41 (0.29−0.57)	1.05 (0.91−1.21)	0.67 (0.48−0.94)	0.58 (0.47−0.71)
≥Secondary education (Primary or less)	0.79 (0.66−0.96)	0.78 (0.62−0.99)	1.12 (0.82−1.53)	1.29 (1.10−1.50)	1.38 (0.97−1.97)	1.01 (0.82−1.25)
Monthly income (USD)						
80−200 (<80)	0.70 (0.55−0.89)	0.77 (0.56−1.06)	0.90 (0.60−1.33)	1.20 (0.99−1.45)	1.11 (0.67−1.83)	0.86 (0.65−1.13)
> 200 (<80)	0.70 (0.56−0.88)	1.03 (0.79−1.34)	1.01 (0.71−1.45)	1.55 (1.31−1.83)	2.24 (1.51−3.33)	1.08 (0.85−1.38)
10+ years on ART (<10 years)	0.85 (0.70−1.02)	0.89 (0.71−1.13)	0.70 (0.52−0.95)	0.66 (0.58−0.77)	0.71 (0.51−1.00)	0.88 (0.7−1.11)
Never diagnosed with AIDS (Ever)	0.92 (0.76−1.13)	0.67 (0.52−0.86)	0.95 (0.68−1.33)	0.90 (0.76−1.06)	1.00 (0.66−1.51)	0.67 (0.54−0.82)

## 4 DISCUSSION

This cross‐sectional analysis of data from PWH aged 40 and older across 11 LMICs enrolled in the IeDEA SRN assessed the prevalence and co‐occurrence of moderate to severe symptoms of depression, anxiety and PTSD, as well as unhealthy alcohol or drug use and their association with socio‐demographic and HIV care factors. Symptoms of mental or substance use disorders were frequently reported with over one‐third (37%) of participants screening positive for any of the mental or substance use outcomes assessed, 18% screening positive for moderate to severe symptoms of depression, anxiety or PTSD and 24% screening positive for unhealthy alcohol or drug use. Psychiatric multimorbidity was also common with 31% of individuals who screened positive for any mental health or substance use outcome assessed having screened positive for more than one.

Our findings are broadly consistent with previous estimates of symptoms of depression [10, 61], anxiety [62], PTSD [63], unhealthy alcohol use [61, 64] and unhealthy drug use [65] among adult PWH across resource‐constrained settings. However, prior estimates of the prevalence of symptoms of depression, anxiety, PTSD and unhealthy substance use specifically among PWH age 40 or older remain limited, particularly in resource‐constrained settings [66]. A review of PWH aged 50 and older in sub‐Saharan Africa found prevalence estimates of depression ranging from 6% to 59%, but study heterogeneity made characterizing mental health challenges among this group difficult [66]. A study among older PWH in Brazil estimated that 28% of participants screened positive for depression which is relatively similar to the 32% of participants in the current analysis from Brazil who screened positive for depressive symptoms [67]. A study conducted in Cote d'Ivoire and Senegal among PWH aged 50 and older found a prevalence of severe depressive symptoms of 18%, similar to the 16% of participants from Cote d'Ivoire in the current analysis who screened positive for depressive symptoms [68]. Caution is warranted when comparing current findings with previous research as the current study began enrolment in October of 2020, during the COVID‐19 pandemic. Previous research has found the burden of mental health disorders increased during the pandemic, including among PWH [69]. Our findings suggest that MSDs remain a significant concern among PWH aged 40 or older across resource‐constrained settings. As the population of older PWH continues to increase, screening and treatment of MSDs among ageing PWH in resource‐constrained settings should be prioritized. The integration of evidence‐based screening and treatment for MSDs into HIV care has the potential to improve access to and uptake of these services.

Psychiatric multimorbidity was also common among this study population, with a third of those who screened positive for one of the mental health or substance use outcomes assessed screening positive for more than one mental health or substance use outcome assessed. Indeed, more than half of individuals who screened positive for moderate to severe symptoms of depression, anxiety, PTSD or unhealthy drug use also screened positive for at least one other mental health or substance use outcome assessed. Prior research into the prevalence of psychiatric multimorbidity among PWH aged 40 or older in resource‐constrained settings remains limited. However, a study of depression, anxiety and stress in Malaysia among ageing PWH (aged ≥25, median age 43), similarly found that multiple conditions were common [70]. Our findings are consistent with prior research among PWH in sub‐Saharan Africa which has estimated the prevalence of psychiatric multimorbidity among PWH with symptoms of at least one mental disorder to be between 18% and 42% [19, 71, 72, 73]. Our findings are also aligned with syndemic‐focused research with PWH which has found that unmet mental health and substance use needs are commonly co‐occurring among PWH in LMICs [74, 75, 76]. Given the prevalence of psychiatric multimorbidity among this population, the implementation and integration of transdiagnostic treatment approaches into HIV care settings should be prioritized. Transdiagnostic treatment approaches may yield efficiencies in the delivery of mental healthcare critical for comprehensively meeting the mental healthcare needs of ageing PWH [77, 78, 79]. The effectiveness of such interventions among older PWH across global settings should be evaluated.

The prevalence of unhealthy alcohol and drug use and symptoms of depression differed meaningfully by gender. Specifically, the prevalence of moderate to severe symptoms of depression and anxiety was higher among women than men, while the prevalence of unhealthy alcohol use and unhealthy drug use was higher among men than women. This is consistent with previous research that has found depression to be more prevalent among women than men in the general population and among PWH and alcohol and drug use to be more prevalent among men than women among the general population and among PWH [10, 64, 80, 81]. Similarly, a study of older (aged 45 or older) individuals (with and without HIV) in Malawi found that depressive symptoms were more prevalent among women than men [82]. Additional research is needed to understand gender‐specific risk and protective factors for MSDs among ageing PWH across resource‐constrained settings.

Age was negatively associated with the prevalence of moderate to severe symptoms of anxiety, PTSD, unhealthy alcohol use and psychiatric multimorbidity in this study, particularly among those 60 years or older. Research in the general population has found that the prevalence of substance use decreases with age [83]. However, the relationship between age and symptoms of MSDs among older PWH remains unclear [10, 65, 84]. For example, in contrast to the current study, a study of older individuals in Malawi (with and without HIV) found that the prevalence of depression and anxiety increased with age [82]. Longitudinal research to examine whether the prevalence of MSDs changes with age among PWH is warranted.

Individuals who were married or partnered had lower prevalence of moderate to severe symptoms of depression, anxiety, PTSD and unhealthy drug use compared to people who were not married or partnered. Similar findings have been found among other populations of PWH [85]. Ageing PWH who were married or partnered may have more robust social support networks and were less socially isolated compared to their unmarried peers. Such greater social support could help explain their better mental health [86]. Research has found that older PWH experience greater social isolation and loss of social networks compared to their ageing peers without HIV [87]. Future research should examine factors that influence the association between relationship status and prevalence of depression, anxiety, PTSD and unhealthy drug use among older PWH in resource‐constrained settings.

The prevalence of symptoms of all MSDs assessed varied substantially by country. The prevalence of moderate to severe symptoms of depression or anxiety was less than 5% in India, Kenya and Vietnam and greater than 25% among participants in Brazil. Similarly, the prevalence of unhealthy alcohol use ranged from 2% in India to 32% in Brazil. It is important to note that data were collected from participants at a single HIV clinic per country. Thus, findings are not representative of the broader population of PWH aged 40 or older in each country. Additional research is needed to better understand the extent to which the prevalence of symptoms of MSDs varies by country among ageing PWH.

Our study's limitations include that the study sample was of PWH age 40 or older who had initiated ART at least 6 months prior to study enrolment. As such, our findings may not be generalizable to the general population of older PWH, including those not yet or newly diagnosed or newly engaged in or disengaged from HIV care. In addition, participants were recruited from a single HIV treatment site in each country. Thus, findings are not generalizable to the general population of PWH aged 40 or older in that country. In addition, mental and substance use screening measures, not diagnostic measures, were to assess mental and substance use symptoms. Screening positive on these measures should not be interpreted as evidence of a clinical disorder [88, 89]. Mental health and substance use assessment tools used in this study were selected because they have been widely used and validated across several populations, languages, countries and with PWH. However, some tools have not been validated in the languages and countries in which they were used.

Our analyses found a substantial burden of symptoms of common mental disorders and unhealthy substance use among PWH aged 40 or older across resource‐constrained settings. This work contributes to a growing understanding of the needs of a population ageing with HIV. Additional research is needed to identify the extent to which risk and protective factors of poor mental health and unhealthy substance use identified in other populations, including disclosure, social support, coping strategies, HIV‐related stigma and social isolation, are relevant among ageing PWH across global settings. In addition, future research should investigate the extent to which issues of particular concern to ageing PWH, including social isolation, co‐occurring NCDs such as diabetes and cardiovascular disease and neurocognitive disorders, influence the prevalence and persistence of symptoms of MSDs among ageing PWH in LMICs. Strategies to promote successful ageing among PWH should incorporate evidence‐based mental health and substance use screening and treatment.

## 5 CONCLUSIONS

In our global cohort of PWH aged 40 and older across LMICs, unhealthy alcohol use and symptoms of depression were most commonly reported. Integration of MSD screening and treatment into HIV care should be prioritized.

## COMPETING INTERESTS

The authors declare no competing interests.

## AUTHORS’ CONTRIBUTIONS

AMP, KL, FM and JR contributed to the design of the survey. AMP, MS and KL contributed to data analysis and interpretation. AMP and MS drafted the manuscript. AMP, MS, CB, TK, EKK, AL, GM, JR, AM, FM, MT, SG, HP, FM, SN and KL revised the manuscript. All authors read and approved the final manuscript.

## FUNDING

The International Epidemiology Databases to Evaluate AIDS (IeDEA) is supported by the U.S. National Institutes of Health's National Institute of Allergy and Infectious Diseases, the *Eunice Kennedy Shriver* National Institute of Child Health and Human Development, the National Cancer Institute, the National Institute of Mental Health, the National Institute on Drug Abuse, the National Heart, Lung, and Blood Institute, the National Institute on Alcohol Abuse and Alcoholism, the National Institute of Diabetes and Digestive and Kidney Diseases, and the Fogarty International Center: ***Asia‐Pacific***, U01AI069907; ***CCASAnet***, U01AI069923; ***Central Africa***, U01AI096299; ***East Africa***, U01AI069911; ***NA‐ACCORD***, U01AI069918; ***Southern Africa***, U01AI069924; ***West Africa***, U01AI069919. Informatics resources are supported by the Harmonist project, R24AI24872.

## DISCLAIMER

This work is solely the responsibility of the authors and does not necessarily represent the official views of any of the institutions mentioned above.

## Supporting information




Table S1: Prevalence and co‐occurrence of symptoms of depression, anxiety, PTSD, or unhealthy alcohol or drug use, Women
Table S2: Prevalence and co‐occurrence of elevated symptoms of mental and substance use disorders, men
**Table S3: Prevalence and co‐occurrence of symptoms of depression, anxiety, PTSD, or unhealthy alcohol or drug use, age 40**−**49**
Table S4: Prevalence and co‐occurrence of symptoms of depression, anxiety, PTSD, or unhealthy alcohol or drug use, age ≥ 50
**Table S5: Prevalence and co‐occurrence of symptoms of depression, anxiety, PTSD, or unhealthy alcohol or drug use,** <**10 years on ART**
Table S6: Prevalence and co‐occurrence of symptoms of depression, anxiety, PTSD, or unhealthy alcohol or drug use, >10 years on ART
Table S7: Prevalence and co‐occurrence of symptoms of depression, anxiety, PTSD, or unhealthy alcohol or drug use, single, divorced, separated, or widowed
Table S8: Prevalence and co‐occurrence of symptoms of depression, anxiety, PTSD, or unhealthy alcohol or drug use, married or living with a partner
Table S9: Prevalence and co‐occurrence of symptoms of depression, anxiety, PTSD, or unhealthy alcohol or drug use, primary or less education
Table S10: Prevalence and co‐occurrence of symptoms of depression, anxiety, PTSD, or unhealthy alcohol or drug use, secondary education or more

## Data Availability

Complete data for this study cannot be posted in a supplemental file or a public repository because of legal and ethical restrictions. The Principles of Collaboration under which the IeDEA multiregional collaboration was founded and the regulatory requirements of the different countries' IRBs require the submission of a project concept proposal and approval by the IeDEA Executive Committee. To request data, please review IeDEA guidance and complete the concept proposal template, available at: https://www.iedea.org/resources/multiregional‐research‐sops‐templates/. Signing of a data‐sharing agreement may also be required.
