# Supplementary material for: Prevalence and co‐occurrence of symptoms of mental and substance use disorders among people with HIV age 40 and older in low‐ and middle‐income countries: a cross‐sectional study
Source: J Int AIDS Soc. 2024 Oct 7;27(10):e26359. doi: 10.1002/jia2.26359 (PMC11458263; doi:10.1002/jia2.26359)
Supplement: Supplementary file 1 — Table S1: Prevalence and co‐occurrence of symptoms of depression, anxiety, PTSD, or unhealthy alcohol or drug use, Women Table S2: Prevalence and co‐occurrence of elevated symptoms of mental and substance use disorders, men Table S3: Prevalence and co‐occurrence of symptoms of depression, anxiety, PTSD, or unhealthy alcohol or drug use, age 40−49 Table S4: Prevalence and co‐occurrence of symptoms of depression, anxiety, PTSD, or unhealthy alcohol or drug use, age ≥ 50 Table S5: Prevalence and co‐occurrence of symptoms of depression, anxiety, PTSD, or unhealthy alcohol or drug use, <10 years on ART Table S6: Prevalence and co‐occurrence of symptoms of depression, anxiety, PTSD, or unhealthy alcohol or drug use, >10 years on ART Table S7: Prevalence and co‐occurrence of symptoms of depression, anxiety, PTSD, or unhealthy alcohol or drug use, single, divorced, separated, or widowed Table S8: Prevalence and co‐occurrence of symptoms of depression, anxiety, PTSD, or unhealthy alcohol or drug use, married or living with a partner Table S9: Prevalence and co‐occurrence of symptoms of depression, anxiety, PTSD, or unhealthy alcohol or drug use, primary or less education Table S10: Prevalence and co‐occurrence of symptoms of depression, anxiety, PTSD, or unhealthy alcohol or drug use, secondary education or more [file JIA2-27-e26359-s001.docx]

**Appendix: Prevalence and co-occurrence of symptoms of depression, anxiety, PTSD, or unhealthy alcohol or drug use, by sex, age, years on ART, education, and relationship status**

**Table 1: Prevalence and co-occurrence of symptoms of depression, anxiety, PTSD, or unhealthy alcohol or drug use, Women**

| n (%) | **Total (N=1565)** | **Depression (n=262)** | **Anxiety (n=161)** | **PTSD (n=87)** | **Unhealthy alcohol use (n=235)** | **Unhealthy drug use (n=40)** |
| --- | --- | --- | --- | --- | --- | --- |
| Symptoms of common mental disorders |  |  |  |  |  |  |
| Depression* | 262 (17) |  | 113 (70) | 66 (76) | 53 (23) | 9 (22) |
| Anxiety** | 161 (10) | 113 (43) |  | 57 (65) | 35 (15) | 6 (15) |
| PTSD** | 87 (6) | 66 (25) | 57 (35) |  | 25 (11) | 5 (12) |
| **Symptoms of depression, anxiety, or PTSD** | 328 (21) |  |  |  | 68 (29) | 13 (32) |
| **Number of common mental disorders** |  |  |  |  |  |  |
| 1 | 200 (13) | 137 (52) | 45 (28) | 18 (21) | 34 (14) | 8 (20) |
| 2 | 74 (5) | 71 (27) | 62 (38) | 15 (17) | 23 (10) | 3 (7) |
| 3 | 54 (3) | 54 (21) | 54 (33) | 54 (62) | 11 (5) | 2 (5) |
| Substance use |  |  |  |  |  |  |
| Unhealthy alcohol use*** | 235 (15) | 53 (20) | 35 (22) | 25 (29) |  | 14 (35) |
| Unhealthy drug use | 40 (3) | 9 (3) | 6 (4) | 5 (6) | 14 (6) |  |
| Unhealthy alcohol or drug use | 261 (17) | 59 (22) | 38 (24) | 27 (31) |  |  |
| **Psychiatric multimorbidity** | 174 (11) | 148 (56) | 122 (76) | 78 (90) | 76 (32) | 21 (52) |

*missing n=1; **missing n=2; ***missing n=4; PTSD=post-traumatic stress disorder

**Table 2: Prevalence and co-occurrence of symptoms of depression, anxiety, PTSD, or unhealthy alcohol or drug use, Men**

| n (%) | **Total (N=1256)** | **Depression (n=125)** | **Anxiety (n=101)** | **PTSD (n=72)** | **Unhealthy alcohol use (n=364)** | **Unhealthy drug use (n=92)** |
| --- | --- | --- | --- | --- | --- | --- |
| Symptoms of common mental disorders |  |  |  |  |  |  |
| Depression* | 125 (10) |  | 59 (58) | 43 (60) | 38 (10) | 14 (15) |
| Anxiety** | 101 (8) | 59 (47) |  | 45 (62) | 36 (10) | 16 (17) |
| PTSD** | 72 (6) | 43 (34) | 45 (45) |  | 32 (9) | 15 (16) |
| **Symptoms of depression, anxiety, or PTSD***** | 184 (15) |  |  |  | 63 (17) | 26 (28) |
| **Number of common mental disorders***** |  |  |  |  |  |  |
| 1 | 103 (8) | 56 (45) | 30 (30) | 17 (24) | 33 (9) | 11 (12) |
| 2 | 48 (4) | 36 (29) | 38 (38) | 22 (31) | 17 (5) | 11 (12) |
| 3 | 33 (3) | 33 (26) | 33 (33) | 33 (46) | 13 (4) | 4 (4) |
| Substance use |  |  |  |  |  |  |
| Unhealthy alcohol use** | 364 (29) | 38 (30) | 36 (36) | 32 (44) |  | 42 (46) |
| Unhealthy drug use*** | 92 (7) | 14 (11) | 16 (16) | 15 (21) | 42 (11) |  |
| Unhealthy alcohol or drug use | 414 (33) | 45 (36) | 45 (45) | 37 (51) |  |  |
|  |  |  |  |  |  |  |
| **Psychiatric multimorbidity** | 149 (12) | 85 (68) | 83 (82) | 66 (92) | 92 (25) | 55 (60) |

*missing n=3; **missing n=2; ***missing n=1; PTSD=post-traumatic stress disorder

**Table 3: Prevalence and co-occurrence of symptoms of depression, anxiety, PTSD, or unhealthy alcohol or drug use, age 40-49**

| n (%) | **Total (N=1375)** | **Depression (n=193)** | **Anxiety (n=139)** | **PTSD (n=88)** | **Unhealthy alcohol use (n=321)** | **Unhealthy drug use (n=72)** |
| --- | --- | --- | --- | --- | --- | --- |
| Symptoms of common mental disorders |  |  |  |  |  |  |
| Depression* | 193 (14) |  | 94 (68) | 61 (69) | 52 (16) | 15 (21) |
| Anxiety** | 139 (10) | 94 (49) |  | 58 (66) | 43 (13) | 16 (22) |
| PTSD** | 88 (6) | 61 (32) | 58 (42) |  | 36 (11) | 17 (24) |
| **Symptoms of depression, anxiety, or PTSD** | 256 (19) |  |  |  | 77 (24) | 27 (37) |
| **Number of common mental disorders** |  |  |  |  |  |  |
| 1 | 141 (10) | 87 (45) | 36 (26) | 18 (20) | 38 (12) | 11 (15) |
| 2 | 66 (5) | 57 (29) | 54 (39) | 21 (24) | 24 (7) | 11 (15) |
| 3 | 49 (4) | 49 (25) | 49 (35) | 49 (56) | 15 (5) | 5 (7) |
| Substance use |  |  |  |  |  |  |
| Unhealthy alcohol use | 321 (23) | 52 (27) | 43 (31) | 36 (41) |  | 34 (47) |
| Unhealthy drug use | 72 (5) | 15 (8) | 16 (11) | 17 (19) | 34 (11) |  |
| Unhealthy alcohol or drug use | 359 (26) | 59 (31) | 50 (36) | 42 (48) |  |  |
| **Psychiatric multimorbidity** | 177 (13) | 126 (65) | 112 (81) | 84 (95) | 96 (30) | 46 (64) |

*missing n=1; **missing n=2; PTSD=post-traumatic stress disorder

**Table 4: Prevalence and co-occurrence of symptoms of depression, anxiety, PTSD, or unhealthy alcohol or drug use, age ≥ 50**

| n (%) | **Total (N=1446)** | **Depression (n=194)** | **Anxiety (n=123)** | **PTSD (n=71)** | **Unhealthy alcohol use (n=278)** | | **Unhealthy drug use (n=60)** |
| --- | --- | --- | --- | --- | --- | --- | --- |
| Symptoms of common mental disorders |  |  |  |  |  |  | |
| Depression* | 194 (13) |  | 78 (63) | 48 (68) | 39 (14) | 8 (13) | |
| Anxiety* | 123 (8) | 78 (40) |  | 44 (62) | 28 (10) | 6 (10) | |
| PTSD** | 71 (5) | 48 (25) | 44 (36) |  | 21 (8) | 3 (5) | |
| **Symptoms of depression, anxiety, or PTSD**** | 256 (18) |  |  |  | 54 (19) | 12 (20) | |
| **Number of common mental disorders**** |  |  |  |  |  |  | |
| 1 | 162 (11) | 106 (55) | 39 (32) | 17 (24) | 29 (10) | 8 (13) | |
| 2 | 56 (4) | 50 (26) | 46 (37) | 16 (22) | 16 (6) | 3 (5) | |
| 3 | 38 (3) | 38 (20) | 38 (31) | 38 (53) | 9 (3) | 1 (2) | |
| Substance use |  |  |  |  |  |  | |
| Unhealthy alcohol use*** | 278 (19) | 39 (20) | 28 (23) | 21 (30) |  | 22 (37) | |
| Unhealthy drug use* | 60 (4) | 8 (4) | 6 (5) | 3 (4) | 22 (8) |  | |
| Unhealthy alcohol or drug use | 316 (22) | 45 (23) | 33 (27) | 22 (31) |  |  | |
| **Psychiatric multimorbidity** | 146 (10) | 107 (55) | 93 (76) | 60 (84) | 72 (26) | 30 (50) | |

*missing n=2; **missing n=1; ***missing n=4; PTSD=post-traumatic stress disorder

**Table 5: Prevalence and co-occurrence of symptoms of depression, anxiety, PTSD, or unhealthy alcohol or drug use,** <**10 years on ART**

| n (%) | **Total (N=1072)** | **Depression (n=162)** | **Anxiety (n=107)** | **PTSD (n=74)** | **Unhealthy alcohol use (n=285)** | **Unhealthy drug use (n=60)** |
| --- | --- | --- | --- | --- | --- | --- |
| Symptoms of common mental disorders |  |  |  |  |  |  |
| Depression* | 162 (15) |  | 70 (65) | 46 (62) | 51 (18) | 14 (23) |
| Anxiety** | 107 (10) | 70 (43) |  | 45 (61) | 38 (13) | 11 (18) |
| PTSD* | 74 (7) | 46 (29) | 45 (42) |  | 33 (12) | 11 (18) |
| **Symptoms of depression, anxiety, or PTSD**** | 217 (20) |  |  |  | 74 (26) | 21 (35) |
| **Number of common mental disorders**** |  |  |  |  |  |  |
| 1 | 126 (12) | 81 (50) | 27 (25) | 18 (24) | 38 (13) | 9 (15) |
| 2 | 56 (5) | 46 (28) | 45 (42) | 21 (28) | 24 (8) | 9 (15) |
| 3 | 35 (3) | 35 (22) | 35 (33) | 35 (47) | 12 (4) | 3 (5) |
| Substance use |  |  |  |  |  |  |
| Unhealthy alcohol use*** | 285 (27) | 51 (31) | 38 (35) | 33 (45) |  | 25 (42) |
| Unhealthy drug use* | 60 (6) | 14 (9) | 11 (10) | 11 (15) | 25 (9) |  |
| Unhealthy alcohol or drug use | 320 (30) | 61 (38) | 45 (42) | 39 (53) |  |  |
| **Psychiatric multimorbidity** | 154 (14) | 106 (65) | 88 (82) | 68 (92) | 92 (32) | 39 (65) |

*missing n=3; **missing n=1; ***missing n=2; PTSD=post-traumatic stress disorder

**Table 6: Prevalence and co-occurrence of symptoms of depression, anxiety, PTSD, or unhealthy alcohol or drug use, >10 years on ART**

| n (%) | **Total (N=1727)** | **Depression (n=222)** | **Anxiety (n=154)** | **PTSD (n=84)** | **Unhealthy alcohol use (n=305)** | **Unhealthy drug use (n=69)** |
| --- | --- | --- | --- | --- | --- | --- |
| Symptoms of common mental disorders |  |  |  |  |  |  |
| Depression | 222 (13) |  | 101 (66) | 63 (75) | 39 (13) | 9 (13) |
| Anxiety* | 154 (9) | 101 (46) |  | 57 (68) | 32 (10) | 11 (16) |
| PTSD** | 84 (5) | 63 (28) | 57 (37) |  | 23 (7) | 9 (13) |
| **Symptoms of depression, anxiety, or PTSD** | 291 (17) |  |  |  | 55 (18) | 18 (26) |
| **Number of common mental disorders** |  |  |  |  |  |  |
| 1 | 174 (10) | 110 (49) | 48 (31) | 16 (19) | 28 (9) | 10 (14) |
| 2 | 65 (4) | 60 (27) | 54 (35) | 16 (19) | 15 (5) | 5 (7) |
| 3 | 52 (3) | 52 (23) | 52 (34) | 52 (62) | 12 (4) | 3 (4) |
| Substance use |  |  |  |  |  |  |
| Unhealthy alcohol use*** | 305 (18) | 39 (18) | 32 (21) | 23 (27) |  | 30 (43) |
| Unhealthy drug use | 69 (4) | 9 (4) | 11 (7) | 9 (11) | 30 (10) |  |
| Unhealthy alcohol or drug use | 344 (20) | 42 (19) | 37 (24) | 24 (29) |  |  |
| **Psychiatric multimorbidity** | 166 (10) | 126 (57) | 116 (75) | 75 (89) | 73 (24) | 36 (52) |

*missing n=3; **missing n=1; ***missing n=2; PTSD=post-traumatic stress disorder

**Table 7: Prevalence and co-occurrence of symptoms of depression, anxiety, PTSD, or unhealthy alcohol or drug use, not married or living with a partner**

| n (%) | **Total (N=1345)** | **Depression (n=249)** | **Anxiety (n=162)** | **PTSD (n=110)** | **Unhealthy alcohol use (n=278)** | **Unhealthy drug use (n=76)** |
| --- | --- | --- | --- | --- | --- | --- |
| Symptoms of common mental disorders |  |  |  |  |  |  |
| Depression* | 249 (18) |  | 114 (70) | 82 (74) | 63 (23) | 19 (25) |
| Anxiety* | 162 (12) | 114 (46) |  | 73 (66) | 42 (15) | 17 (22) |
| PTSD** | 110 (8) | 82 (33) | 73 (45) |  | 41 (15) | 15 (20) |
| **Symptoms of depression, anxiety, or PTSD** | 316 (23) |  |  |  | 81 (29) | 28 (37) |
| **Number of common mental disorders** |  |  |  |  |  |  |
| 1 | 175 (13) | 117 (47) | 39 (24) | 19 (17) | 36 (13) | 11 (14) |
| 2 | 77 (6) | 68 (27) | 59 (36) | 27 (24) | 25 (9) | 11 (14) |
| 3 | 64 (5) | 64 (26) | 64 (39) | 64 (58) | 20 (7) | 6 (8) |
| Substance use |  |  |  |  |  |  |
| Unhealthy alcohol use*** | 278 (21) | 63 (25) | 42 (26) | 41 (37) |  | 28 (37) |
| Unhealthy drug use* | 76 (6) | 19 (8) | 17 (10) | 15 (14) | 28 (10) |  |
| Unhealthy alcohol or drug use | 326 (24) | 73 (29) | 53 (33) | 46 (42) |  |  |
| **Psychiatric multimorbidity** | 198 (15) | 157 (63) | 129 (80) | 102 (93) | 96 (34) | 43 (57) |

*missing n=2; **missing n=1; ***missing n=3; PTSD=post-traumatic stress disorder

**Table 8: Prevalence and co-occurrence of symptoms of depression, anxiety, PTSD, or unhealthy alcohol or drug use, married or living with a partner**

| n (%) | **Total (N=1472)** | **Depression (n=138)** | **Anxiety (n=100)** | **PTSD (n=49)** | **Unhealthy alcohol use (n=320)** | **Unhealthy drug use (n=56)** |
| --- | --- | --- | --- | --- | --- | --- |
| Symptoms of common mental disorders |  |  |  |  |  |  |
| Depression* | 138 (9) |  | 58 (58) | 27 (55) | 28 (9) | 4 (7) |
| Anxiety** | 100 (7) | 58 (42) |  | 29 (59) | 29 (9) | 5 (9) |
| PTSD** | 49 (3) | 27 (20) | 29 (29) |  | 16 (5) | 5 (9) |
| **Symptoms of depression, anxiety, or PTSD*** | 196 (13) |  |  |  | 50 (16) | 11 (20) |
| **Number of common mental disorders*** |  |  |  |  |  |  |
| 1 | 128 (9) | 76 (55) | 36 (36) | 16 (33) | 31 (10) | 8 (14) |
| 2 | 45 (3) | 39 (28) | 41 (41) | 10 (20) | 15 (5) | 3 (5) |
| 3 | 23 (2) | 23 (17) | 23 (23) | 23 (47) | 4 (1) | (0) |
| Unhealthy substance use |  |  |  |  |  |  |
| Unhealthy alcohol use* | 320 (22) | 28 (20) | 29 (29) | 16 (33) |  | 28 (50) |
| Unhealthy drug use | 56 (4) | 4 (3) | 5 (5) | 5 (10) | 28 (9) |  |
| Unhealthy alcohol or drug use | 348 (24) | 31 (22) | 30 (30) | 18 (37) |  |  |
| **Psychiatric multimorbidity** | 125 (8) | 76 (55) | 76 (76) | 42 (86) | 72 (22) | 33 (59) |

*missing n=1; **missing n=2; PTSD=post-traumatic stress disorder

**Table 9: Prevalence and co-occurrence of symptoms of depression, anxiety, PTSD, or unhealthy alcohol or drug use, primary education or less**

| n (%) | **Total (N=1119)** | **Depression (n=175)** | **Anxiety (n=119)** | **PTSD (n=59)** | **Unhealthy alcohol use (n=203)** | **Unhealthy drug use (n=43)** |
| --- | --- | --- | --- | --- | --- | --- |
| Symptoms of common mental disorders |  |  |  |  |  |  |
| Depression* | 175 (16) |  | 75 (63) | 48 (81) | 33 (16) | 9 (21) |
| Anxiety* | 119 (11) | 75 (43) |  | 40 (68) | 22 (11) | 8 (19) |
| PTSD* | 59 (5) | 48 (28) | 40 (34) |  | 12 (6) | 4 (9) |
| **Symptoms of depression, anxiety, or PTSD**** | 228 (20) |  |  |  | 45 (22) | 15 (35) |
| **Number of common mental disorders**** |  |  |  |  |  |  |
| 1 | 141 (13) | 90 (51) | 42 (35) | 9 (15) | 27 (13) | 10 (23) |
| 2 | 49 (4) | 47 (27) | 39 (33) | 12 (20) | 14 (7) | 4 (9) |
| 3 | 38 (3) | 38 (22) | 38 (33) | 38 (64) | 4 (2) | 1 (2) |
| Substance use |  |  |  |  |  |  |
| Unhealthy alcohol use | 203 (18) | 33 (19) | 22 (18) | 12 (20) |  | 14 (33) |
| Unhealthy drug use | 43 (4) | 9 (5) | 8 (7) | 4 (7) | 14 (7) |  |
| Unhealthy alcohol or drug use | 232 (21) | 40 (23) | 27 (23) | 14 (24) |  |  |
| **Psychiatric multimorbidity** | 127 (11) | 104 (59) | 86 (72) | 54 (91) | 53 (26) | 23 (53) |

*missing n=2; **missing n=1; PTSD=post-traumatic stress disorder

**Table 10: Prevalence and co-occurrence of symptoms of depression, anxiety, PTSD, or unhealthy alcohol or drug use, secondary education or greater**

| n (%) | **Total (N=1680)** | **Depression (n=209)** | **Anxiety (n=140)** | **PTSD (n=99)** | **Unhealthy alcohol use (n=391)** | **Unhealthy drug use (n=89)** |
| --- | --- | --- | --- | --- | --- | --- |
| Symptoms of common mental disorders |  |  |  |  |  |  |
| Depression* | 209 (12) |  | 95 (68) | 60 (61) | 58 (15) | 14 (16) |
| Anxiety** | 140 (8) | 95 (46) |  | 61 (62) | 48 (12) | 14 (16) |
| PTSD* | 99 (6) | 60 (29) | 61 (44) |  | 45 (11) | 16 (18) |
| **Symptoms of depression, anxiety, or PTSD** | 280 (17) |  |  |  | 85 (22) | 24 (27) |
| **Number of common mental disorders** |  |  |  |  |  |  |
| 1 | 160 (9) | 102 (49) | 32 (23) | 26 (26) | 39 (10) | 9 (10) |
| 2 | 72 (4) | 59 (28) | 60 (43) | 25 (25) | 26 (7) | 10 (11) |
| 3 | 48 (3) | 48 (23) | 48 (34) | 48 (48) | 20 (5) | 5 (6) |
| Substance use |  |  |  |  |  |  |
| Unhealthy alcohol use*** | 391 (23) | 58 (28) | 48 (34) | 45 (45) |  | 42 (47) |
| Unhealthy drug use* | 89 (5) | 14 (7) | 14 (10) | 16 (16) | 42 (11) |  |
| Unhealthy alcohol or drug use | 438 (26) | 64 (31) | 55 (39) | 50 (50) |  |  |
| **Psychiatric multimorbidity** | 193 (11) | 127 (61) | 116 (83) | 89 (90) | 114 (29) | 53 (60) |

*missing n=1; **missing n=2; ***missing n-3; PTSD=post-traumatic stress disorder
